# Antidiabetic and lipid-lowering therapy modify the association between triglyceride–glucose index and acute kidney injury in critically ill patients with coronary artery disease

**DOI:** 10.3389/fendo.2025.1699936

**Published:** 2025-11-21

**Authors:** Yuehang Yang, Linfeng He, Xueying Jiao, Hanshen Luo, Xiang Qiu, Chuli Shi, Li Ding, Jiawei Shi

**Affiliations:** 1 Department of Cardiovascular Surgery, Union Hospital, Tongji Medical College, Huazhong University of Science and Technology, Wuhan, Hubei, China; 2 Department of Endocrinology and Metabolism, The Second Affiliated Hospital, Jiangxi Medical College, Nanchang University, Nanchang, China; 3 Department of Global Public Health, Karolinska Institutet, Stockholm, Sweden

**Keywords:** triglyceride-glucose index, coronary heart disease, acute kidney injury, antidiabetic drugs, lipid-lowering drugs

## Abstract

**Background:**

Acute kidney injury (AKI) is a common and serious complication in critically ill coronary artery disease (CAD) patients. The triglyceride-glucose (TyG) index, a surrogate marker of insulin resistance, has been linked to adverse cardiovascular and renal outcomes. However, whether antidiabetic drugs or lipid-lowering drugs modify its association with AKI in this population remains unclear.

**Methods:**

This study retrospectively analyzed 2,517 critically ill CAD patients from the MIMIC-IV database. Patients were stratified according to the use of antidiabetic and lipid-lowering drugs. The primary endpoint was the occurrence of AKI during hospitalization. Multivariable logistic regression and restricted cubic spline (RCS) models were applied to examine the association between the TyG index and AKI risk. Subgroup analyses, sensitivity analyses, and independent external validation were performed to assess the robustness of the findings.

**Results:**

The median age of patients was 69 years, and 68.06% were male. In the fully adjusted logistic regression model, a higher TyG index was significantly associated with an increased risk of AKI among patients without the use of antidiabetic drugs (OR 2.021, 95% CI 1.674–2.454) or lipid-lowering drugs (OR 1.912, 95% CI 1.648–2.228). With the use of antidiabetic drugs, this association remained significant but was attenuated (OR 1.480, 95% CI 1.190–1.853), with a significant interaction observed between the use of antidiabetic drugs and the TyG index in relation to AKI risk (P for interaction = 0.040). With the use of lipid-lowering drugs, the association between the TyG index and AKI risk was weakened (OR 1.445, 95% CI 0.934–2.307), but no significant interaction was found (P for interaction = 0.332). RCS analyses demonstrated a linear relationship between higher TyG index values and increased AKI risk. Similar results were confirmed in external validation.

**Conclusions:**

In critically ill CAD patients, a higher TyG index was independently associated with an increased risk of AKI, whereas this association was attenuated in those with the use of antidiabetic or lipid-lowering drugs. These findings highlight the importance of incorporating metabolic risk assessment into the management of critically ill patients and underscore the potential of pharmacological interventions to improve renal outcomes.

## Introduction

Acute kidney injury (AKI), a common and serious complication in patients with coronary artery disease (CAD), is closely associated with adverse cardiovascular events and increased mortality among those admitted to the intensive care unit (ICU) (1). Heart failure can impair renal function by affecting metabolic or fluid regulation, leading to cardiorenal syndrome and increasing susceptibility to sepsis, respiratory failure, and other complications (2, 3). These observations highlight the urgent need for early risk stratification in CAD patients to improve clinical outcomes.

Insulin resistance (IR) is primarily characterized by reduced insulin sensitivity, which contributes to vascular stiffening and the development of cardiovascular disease (4). The triglyceride-glucose (TyG) index is a simple and reliable surrogate marker of IR, calculated from fasting triglyceride and fasting glucose levels (5). Previous studies have shown that a higher TyG index is closely associated with an increased risk of atherosclerosis, heart failure, hypertension, and diabetes across different populations (6–9). Importantly, growing evidence indicates that metabolic dysregulation, dyslipidemia, and inflammation act synergistically to influence cardiorenal outcomes (10). Excessive lipid accumulation in the circulation disrupts glucose metabolism, which contributes to insulin resistance and pancreatic β-cell dysfunction, ultimately elevating the risk of cardiovascular disease (11). Moreover, obesity and dyslipidemia enhance systemic inflammation, leading to endothelial dysfunction and oxidative stress, thereby aggravating both vascular and renal injury (12, 13). In critically ill patients, the TyG index has demonstrated potential prognostic value for adverse renal outcomes, suggesting it may serve as an early clinical marker for AKI risk assessment (14).

However, the TyG index may be influenced by pharmacological interventions. Antidiabetic and lipid-lowering drugs can alter the TyG index through their effects on glucose and lipid metabolism, potentially modifying its association with cardiovascular disease (15, 16). For example, antidiabetic drugs improve glycemic control, which may mitigate renal injury related to insulin resistance (17). Lipid-lowering drugs not only reduce triglyceride levels but also alleviate inflammation, oxidative stress, and improve endothelial function, thereby exerting beneficial effects on renal function in patients with cardiovascular disease (18). Recent evidence suggests that antidiabetic and lipid-lowering drugs can modify the relationship between the TyG index and cardiovascular mortality (19). However, whether these drugs influence the association between the TyG index and AKI in critically ill CAD patients remains unclear. Therefore, this study aimed to investigate whether the use of antidiabetic and lipid-lowering drugs alters the relationship between the TyG index and AKI risk in critically ill CAD patients, providing targeted strategies for AKI prevention in this high-risk population.

## Methods

### Source of data

This study is a retrospective analysis using data from the publicly available Medical Information Mart for Intensive Care IV (MIMIC-IV, version 3.1) database, which includes hospitalization information for critically ill patients admitted to Beth Israel Deaconess Medical Center between 2008 and 2019. To comply with relevant regulations, the author (Yuehang Yang) obtained access to the database (ID: 59781685) and extracted the data. As all patient information in the database is de-identified to protect privacy, informed consent was not required. Data from our center were obtained through the electronic medical record system. This study was approved by the Ethics Committee of Union Hospital, Tongji Medical College, Huazhong University of Science and Technology, and conducted in accordance with the Declaration of Helsinki. All patient-related data were de-identified to ensure confidentiality.

### Study population

**Figure 1:**
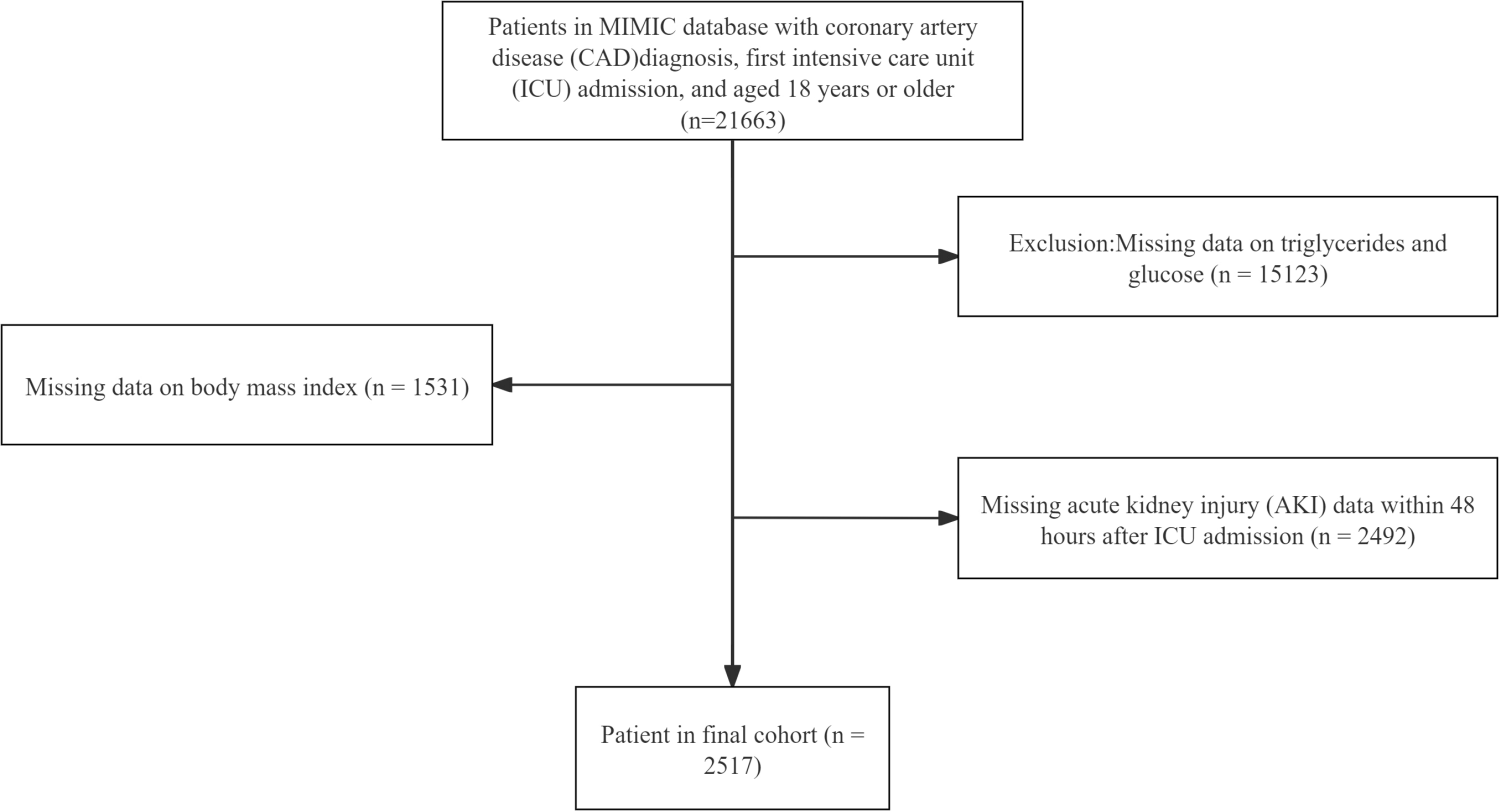
Flowchart of patient selection from the MIMIC-IV database. MIMIC-IV, medical information mart for intensive care-iv.

The study population from MIMIC-IV comprised 21,663 patients diagnosed with CAD who were admitted to the intensive care unit (ICU) for the first time (≥18 years). The exclusion criteria were: (1) missing triglyceride or glucose data, (2) missing body mass index (BMI) data, and (3) missing AKI-related data within 48 hours of ICU admission. After applying these criteria, 2,517 patients were included in the study. (**Figure 1**) Data from our center were retrospectively collected using the same inclusion and exclusion criteria, comprising 910 patients diagnosed with CAD and admitted to the ICU at Union Hospital, Tongji Medical College, Huazhong University of Science and Technology, between November 2021 and June 2024, serving as an external validation cohort.

### Data collection

Data were extracted using Structured Query Language (SQL) with PostgreSQL (version 16.0). Including demographics (age, sex, BMI), laboratory tests (red blood cell [RBC], white blood cell [WBC], platelet [PLT], anion gap, calcium, potassium, sodium, fasting plasma glucose [FPG], international normalized ratio [INR], high-density lipoprotein cholesterol [HDL], low-density lipoprotein cholesterol [LDL], glycated hemoglobin [HbA1c], triglyceride [TG], alanine aminotransferase [ALT], aspartate aminotransferase [AST], total bilirubin, creatinine, urea nitrogen), comorbidities (hypertension, hepatitis, liver cirrhosis, stroke, cancer, hyperlipidemia, chronic obstructive pulmonary disease [COPD], diabetes, heart failure), severity scores on admission (Sequential Organ Failure Assessment [SOFA], Acute Physiology Score III [APS III], Simplified Acute Physiology Score II [SAPS II], Oxford Acute Severity of Illness Score [OASIS], Charlson Comorbidity Index), medication use (β-blockers, angiotensin-converting enzyme inhibitors [ACEI], angiotensin receptor blockers [ARB]), coronary artery bypass grafting (CABG), and percutaneous coronary intervention (PCI). Laboratory measurements were taken at ICU admission. CAD was defined as angina, myocardial infarction, acute coronary syndrome, ischemic cardiomyopathy, or prior CABG. Comorbidities, CABG, and PCI were identified using ICD-9 and ICD-10 codes. At Union Hospital, Tongji Medical College, Huazhong University of Science and Technology, demographics, preoperative laboratory tests, comorbidities, medication use, prior coronary stent implantation, duration of mechanical ventilation, length of hospital stay, and ICU stay were collected. Variables with more than 20% missing data were excluded, and those with less than 20% missing data were imputed using multiple imputation. The TyG index was calculated as follows: TyG = ln [TG (mg/dL)×FPG (mg/dL)/2].

### Endpoints of interest

The primary endpoint was the occurrence of AKI. Globally, AKI is defined as: an increase in serum creatinine by ≥0.3 mg/dL within 48 hours; or an increase in serum creatinine to ≥1.5 times the baseline within the prior 7 days; or urine output <0.5 mL/kg/h for at least 6 hours. The initial creatinine value at admission was used as the baseline.

### Statistical analysis

Both datasets were first divided into tertiles based on the TyG index. In MIMIC-IV, the tertiles were T1 (≤8.74), T2 (8.74–9.36), and T3 (≥9.36), while in the external validation cohort, they were T1 (≤8.76), T2 (8.76–9.38), and T3 (≥9.38). Normality of all continuous variables was assessed, and all P-values were <0.05, indicating non-normal distribution; therefore, the Kruskal–Wallis H test was used for continuous variables, presented as median (interquartile range). Categorical variables were analyzed using the chi-square test or Fisher’s exact test, presented as counts (percentages). Patients were stratified according to the use of antidiabetic and lipid-lowering drugs, and logistic regression models were applied to examine the association between the TyG index and AKI, adjusting for various covariates. Model 1 was unadjusted. Model 2 adjusted for age, sex, and BMI. Model 3 further adjusted for age, sex, BMI, hypertension, stroke, hyperlipidemia, liver cirrhosis, and COPD. Restricted cubic spline (RCS) plots were used based on Model 3 to evaluate the relationship between continuous TyG index values and AKI, highlighting the clinical effects of antidiabetic and lipid-lowering drug use. Subgroup and interaction analyses were performed for age (≤ 70 years vs. >70 years), sex (male vs. female), BMI (≤29.7 vs. >29.7), hypertension, stroke, and heart failure, with results visualized using forest plots. Sensitivity analysis including patients excluded due to missing BMI values was conducted to assess robustness. In the external validation cohort, logistic regression models were applied similarly: Model 1 unadjusted; Model 2 adjusted for age, sex, and BMI; Model 3 further adjusted for age, sex, BMI, hypertension, stroke, and COPD. All analyses were performed using R Studio (version 4.2.3), and two-sided P-values <0.05 were considered statistically significant.

## Results

### Baseline characteristics

The MIMIC-IV cohort included 2,517 participants. As shown in **Supplementary Tables S1–S3**, patients were stratified according to the use of antidiabetic drugs, lipid-lowering drugs, and the presence of AKI. The median age of the entire cohort was 69 years, and 68.06% were male. The median levels of metabolic parameters were as follows: FPG 135.00 mg/dL (110.00–179.00), HDL 41.00 mg/dL (32.00–51.00), LDL 76.00 mg/dL (53.00–107.00), triglycerides 119.00 mg/dL (85.00–180.00), TyG index 9.04 (8.60–9.58), creatinine 1.10 mg/dL (0.80–1.60), and blood urea nitrogen 20.00 mg/dL (15.00–33.00). The prevalence of comorbidities was as follows: stroke (10.37%), hyperlipidemia (53.64%), diabetes mellitus (38.82%), and heart failure (45.29%). In addition, 23.76% of patients underwent coronary artery bypass grafting, and 16.09% underwent percutaneous coronary intervention. Baseline characteristics of the external validation dataset are presented in **Supplementary Tables S4–S6**.

### Associations of TyG index with AKI

**Table 1** and **Table 2** present the unadjusted and adjusted associations of the TyG index with AKI risk stratified by antidiabetic and lipid-lowering drug use. When analyzed as a continuous variable, Model 3 indicated that the TyG index was significantly associated with AKI risk both in patients without antidiabetic drugs (OR: 2.021, 95% CI: 1.674–2.454, *P* < 0.001) and in those with antidiabetic drugs (OR: 1.480, 95% CI: 1.190–1.853, *P* < 0.001). Moreover, compared with T1, T3 of the TyG index was significantly associated with a higher AKI risk in patients without antidiabetic drugs (OR: 2.550, 95% CI: 1.868–3.501, *P* < 0.001) and in those with antidiabetic drugs (OR: 2.218, 95% CI: 1.408–3.550, *P* < 0.001). A significant interaction between antidiabetic drug use and the TyG index was observed (*P* for interaction = 0.040). In contrast, Model 3 demonstrated that the TyG index was significantly associated with AKI risk in patients without lipid-lowering drug use (OR: 1.912, 95% CI: 1.648–2.228, P<0.001), whereas no significant association was observed in those with lipid-lowering drug use. No significant interaction between lipid-lowering drug use and the TyG index was found (*P* for interaction = 0.332), possibly due to the limited number of AKI events in these patients. Similar findings were observed in the external validation dataset, where higher TyG index levels were significantly associated with increased AKI risk in patients without antidiabetic or lipid-lowering drug use, while no significant association was observed in those with such drug use across any models (**Supplementary Tables S7, S8**). The lack of significant interactions may be attributed to insufficient sample size.

**Table 1:** Association between the TyG index and AKI, stratified by antidiabetic drug use.

Categories	Number of incidence (rate)	Model I OR (95%CI)	*P*	Model II OR (95%CI)	*P*	Model III OR (95%CI)	*P*	*P* for interaction
Without antidiabetic drug								0.040
Continuous variable per 1 unit		1.939 (1.628,2.323)	<0.001	1.988 (1.656,2.400)	<0.001	2.021 (1.674,2.454)	<0.001	
Tertile								
TyG index								
T1 (≤8.74)	319 (64.57)	Ref		Ref		Ref		
T2 (8.74-9.36)	355 (71.86)	1.401 (1.071,1.835)	0.014	1.377 (1.046,1.815)	0.023	1.398 (1.053,1.857)	0.021	
T3 (≥9.36)	404 (81.62)	2.435 (1.822,3.273)	<0.001	2.490 (1.839,3.391)	<0.001	2.550 (1.868,3.501)	<0.001	
P for trend			<0.001		<0.001		<0.001	
With antidiabetic drug								
Continuous variable per 1 unit		1.603 (1.306,1.983)	<0.001	1.511 (1.219,1.888)	<0.001	1.480 (1.190,1.853)	<0.001	
Tertile								
TyG index								
T1 (≤8.74)	268 (77.91)	Ref		Ref		Ref		
T2 (8.74-9.36)	273 (79.36)	1.090 (0.757,1.572)	0.642	1.023 (0.704,1.489)	0.904	1.046 (0.717,1.528)	0.814	
T3 (≥9.36)	312 (90.17)	2.602 (1.696,4.066)	<0.001	2.313 (1.479,3.678)	<0.001	2.218 (1.408,3.550)	<0.001	
P for trend			<0.001		<0.001		0.001	

Model I: Unadjusted.

Model II: Adjusted for age, sex, BMI.

Model III: Adjusted for age, sex, BMI, COPD, hypertension, liver cirrhosis, stroke, hyperlipemia.

95% CI, 95% confidence interval; OR, odds ratio; Ref, reference; TyG, triglyceride-glucose.

*P for interactions was calculated for Model III.

**Table 2:** Association between the TyG index and AKI, stratified by lipid-lowering drug use.

Categories	Number of incidence (rate)	Model I OR (95%CI)	*P*	Model II OR (95%CI)	*P*	Model III OR (95%CI)	*P*	*P* for interaction
Without lipid-lowering drug								0.332
Continuous variable per 1 unit		1.908 (1.659,2.202)	<0.001	1.897 (1.640,2.204)	<0.001	1.912 (1.648,2.228)	<0.001	
Tertile								
TyG index								
T1 (≤8.74)	514 (68.53)	Ref		Ref		Ref		
T2 (8.74-9.36)	561 (74.80)	1.363 (1.088,1.709)	0.007	1.327 (1.054,1.672)	0.016	1.360 (1.076,1.720)	0.010	
T3 (≥9.36)	652 (86.93)	3.055 (2.357,3.985)	<0.001	3.024 (2.304,3.993)	<0.001	3.121 (2.368,4.138)	<0.001	
P for trend			<0.001		<0.001		<0.001	
With lipid-lowering drug								
Continuous variable per 1 unit		1.563 (1.062,2.383)	0.029	1.423 (0.943,2.231)	0.105	1.445 (0.934,2.307)	0.109	
Tertile								
TyG index								
T1 (≤8.74)	62 (69.66)	Ref		Ref		Ref		
T2 (8.74-9.36)	68 (76.40)	1.410 (0.726,2.767)	0.312	1.392 (0.695,2.284)	0.354	1.297 (0.627,2.710)	0.485	
T3 (≥9.36)	74 (83.15)	2.148 (1.062,4.481)	0.036	1.842 (0.870,3.996)	0.115	1.835 (0.848,4.059)	0.127	
P for trend			0.036		0.111		0.127	

Model I: Unadjusted.

Model II: Adjusted for age, sex, BMI.

Model III: Adjusted for age, sex, BMI, COPD, hypertension, liver cirrhosis, stroke, hyperlipemia.

95% CI, 95% confidence interval; OR, odds ratio; Ref, reference; TyG, triglyceride-glucose.

*P for interactions was calculated for Model III.

### Restricted cubic spline

**Figure 2:**
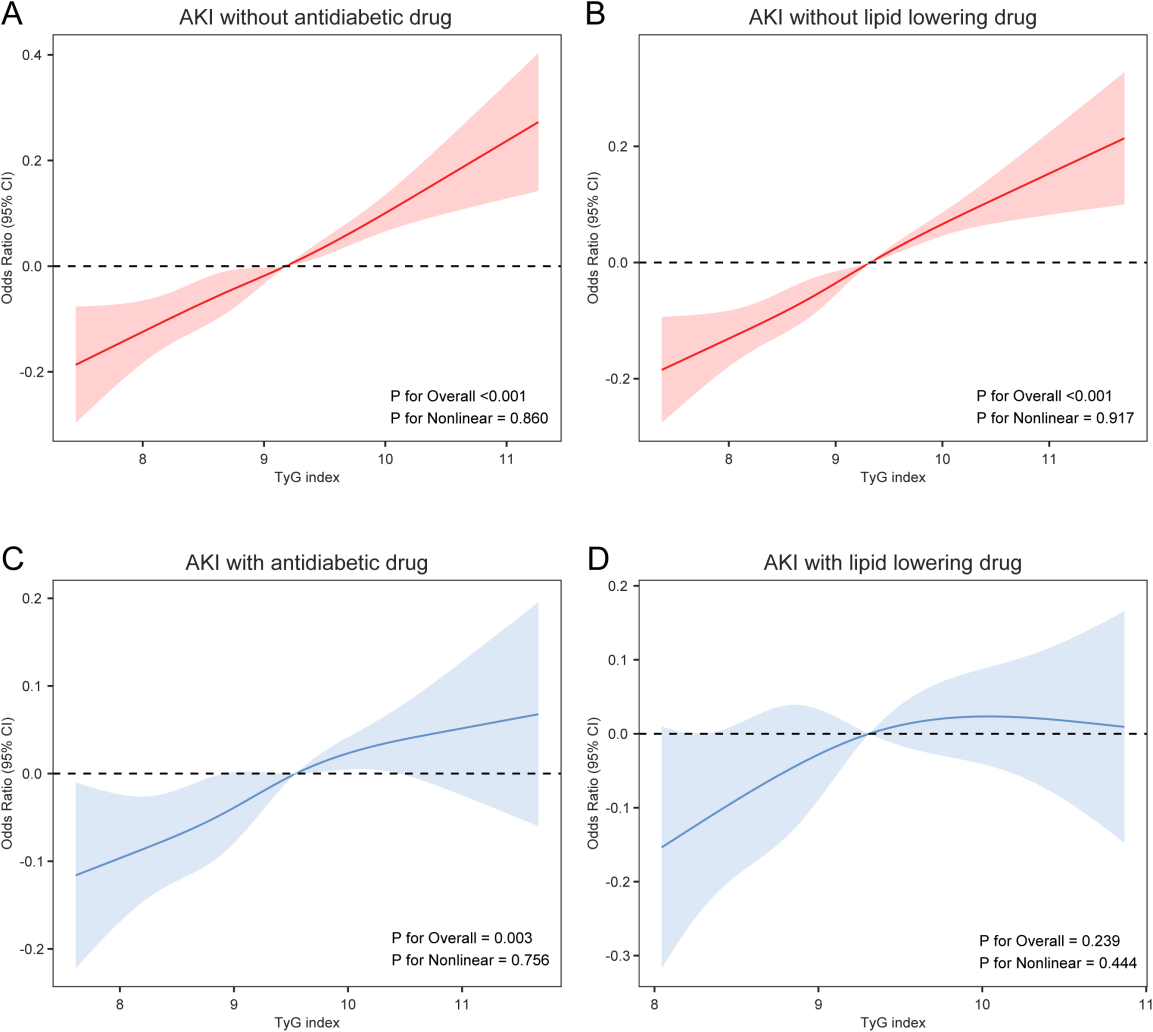
Association of the TyG Index with AKI, stratified by antidiabetic and lipid-lowering drug use. **(A)** Restricted cubic spline curve for the AKI without antidiabetic drug; **(B)** Restricted cubic spline curve for the AKI without lipid lowering drug; **(C)** Restricted cubic spline curve for the AKI with antidiabetic drug; **(D)** Restricted cubic spline curve for the AKI with lipid lowering drug. The non-medication group was indicated in red, while the medication group was indicated in blue, shaded areas indicate 95% CIs. AKI, acute kidney injury; TyG index, triglyceride-glucose index.

RCS analyses showed a linear association between the TyG index and AKI risk (*P* for nonlinearity = 0.755, 0.809, and 0.450) (**Supplementary Figure S1**). This relationship was significant in patients without antidiabetic (*P* for overall < 0.001, *P* for nonlinearity = 0.860) or lipid-lowering drug use (*P* for overall < 0.001, *P* for nonlinearity = 0.917) (**Figures 2A, B**). Among patients using antidiabetic drugs, the association between TyG index and AKI risk persisted (*P* for overall = 0.003, *P* for nonlinearity = 0.756) (**Figure 2C**). In contrast, no significant association was observed in patients using lipid-lowering drugs (*P* for overall = 0.239) (**Figure 2D**). In the external validation cohort, the association between the TyG index and AKI risk was significant among patients without antidiabetic or lipid-lowering drug use (*P* for overall < 0.001), but not significant in those using these two drugs (*P* for overall > 0.05) (**Supplementary Figure S2**).

### Subgroup analysis

**Figure 3:**
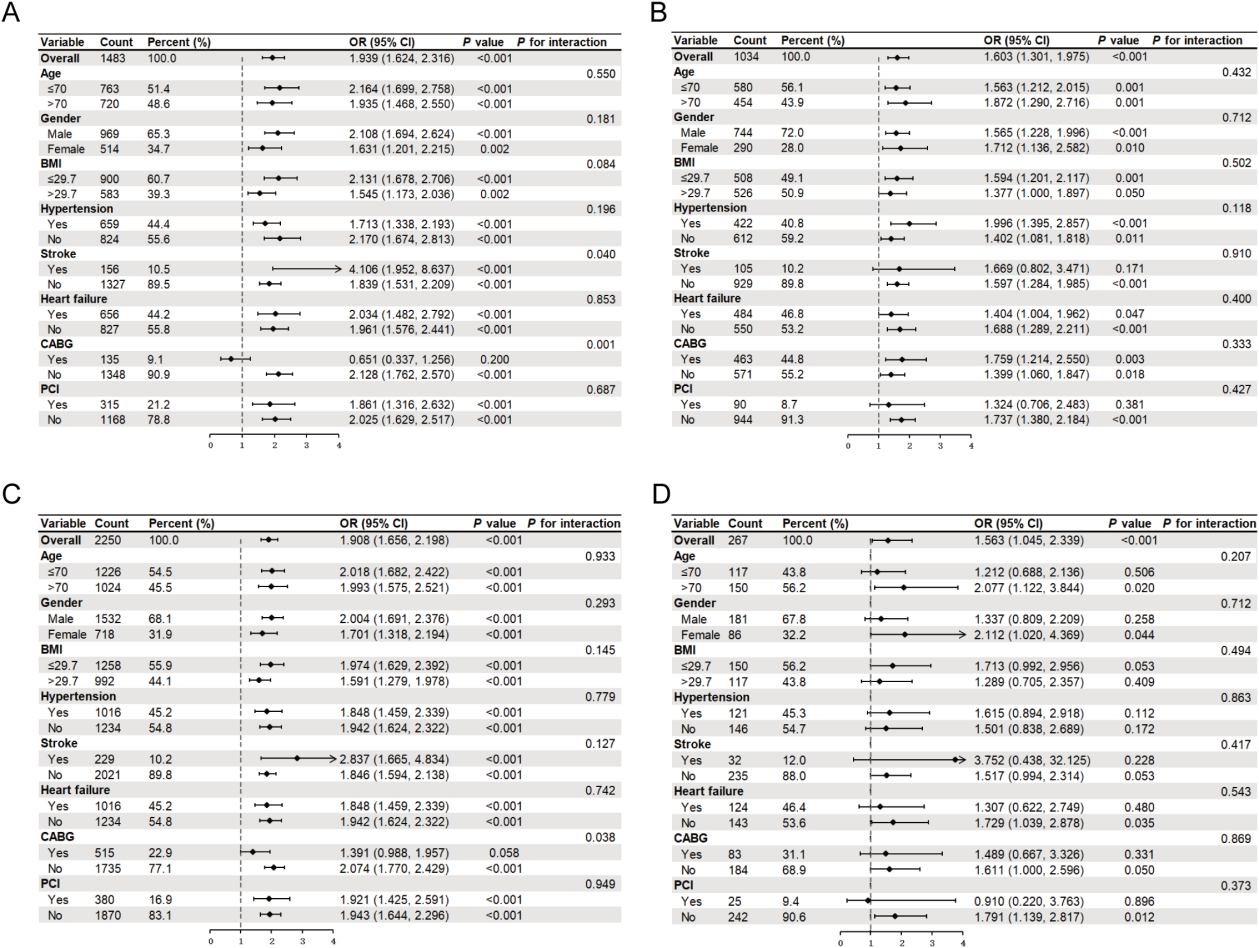
Subgroup analysis forest plot stratified by antidiabetic or lipid-lowering drug use. **(A)** Without antidiabetic drug; **(B)** With antidiabetic drug; **(C)** Without lipid lowering drug; **(D)** With lipid lowering drug. BMI, body mass index; OR, odds ratio; CI, confidence interval.

To further confirm the association between the TyG index and AKI across different subgroups, analyses were stratified by age (≤ 70 or >70 years), sex (male or female), BMI (≤ 29.7 or >29.7), hypertension, stroke, and heart failure. Among patients without antidiabetic or lipid-lowering drug use, the TyG index was significantly associated with AKI in most subgroups (*P* < 0.05) (**Figures 3A, C**). Notably, a significant interaction was observed in the stroke subgroup (*P* for interaction = 0.040) and CABG subgroup (*P* for interaction = 0.001) among patients without antidiabetic drug use. In patients without lipid-lowering drug use, a significant interaction was also observed in the CABG subgroup (*P* for interaction = 0.038). In patients using antidiabetic drugs, the TyG index remained significantly associated with AKI in all subgroups (*P* < 0.05) (**Figure 3B**). In contrast, among patients using lipid-lowering drugs, the association between the TyG index and AKI was weakened, with significant associations observed only in a few subgroups, such as those aged >70 years (*P* = 0.020), female patients (*P* = 0.044), and patients without heart failure (*P* = 0.035) (**Figure 3D**).

### Sensitivity analysis

To further verify the robustness of the results, a sensitivity analysis was performed by including individuals excluded from the MIMIC-IV database due to missing BMI data, using logistic regression. The TyG index was reclassified into tertiles: T1 (≤ 8.70), T2 (8.70–9.30), and T3 (≥ 9.30). The main findings regarding antidiabetic and lipid-lowering drug use were consistent with the previous analyses. Significant interactions were observed between TyG index and antidiabetic drug use (*P* for interaction = 0.004) as well as lipid-lowering drug use (*P* for interaction = 0.008) (**Supplementary Tables S9, S10**).

## Discussion

In this study, we found that higher TyG index levels were significantly associated with an increased risk of AKI in critically ill CHD patients. However, this association was attenuated among patients using antidiabetic or lipid-lowering drugs. Logistic regression analysis indicated that antidiabetic drug use significantly reduced the association between the TyG index and AKI risk. Although the interaction between lipid-lowering drug use and the TyG index was not significant in the initial analysis, the sensitivity analysis with an expanded sample size further demonstrated that lipid-lowering therapy could also significantly weaken this association. The findings from the RCS analysis were consistent with the main results. Taken together, these results suggest that pharmacological modulation of glucose and lipid metabolism may alter the relationship between the TyG index and AKI, providing potential clinical guidance for preventing AKI in patients with CHD.

Previous studies have shown a close association between the TyG index and an increased risk of chronic kidney disease (20). In critically ill patients with hypertension or heart failure, the TyG index has been identified as an independent predictor of AKI and adverse renal outcomes (21, 22). Compared with patients with normal blood glucose levels, hyperglycemia is associated with a higher risk of in-hospital AKI, infections, and stroke (23). Critically ill patients with AKI are at increased risk of hyperglycemia due to impaired renal metabolic function and accumulation of toxins, which can further elevate mortality (24). In our study, by incorporating medication use into the analysis, we further confirmed the association between the TyG index and adverse renal outcomes and proposed that the use of antidiabetic or lipid-lowering drugs may modify the relationship between the TyG index and AKI risk in patients with CAD. A retrospective study reported that metformin use significantly reduced the risk of postoperative AKI in diabetic patients undergoing CABG (25). Similarly, two meta-analyses demonstrated that antidiabetic drugs, particularly those with pleiotropic effects such as sodium-glucose cotransporter 2 (SGLT2) inhibitors, provide renal protection beyond glycemic control, including anti-inflammatory, antioxidant, and hemodynamic benefits (26, 27). Studies have also shown that lipid-lowering therapy exerts renoprotective effects in patients with chronic kidney disease, regardless of diabetes status (28). Recent evidence in familial hypercholesterolemia demonstrates that genotype-specific differences in glycemic homeostasis critically modulate atherosclerosis distribution and cardiorenal vulnerability, underscoring the importance of integrated metabolic–lipid assessment for risk stratification and therapeutic optimization (29). A multicenter clinical trial reported that lipid-lowering drugs effectively prevented AKI in diabetic patients (30). Moreover, emerging lipid-lowering drugs, such as inclisiran, may offer a safer and more effective means of reducing the risk of cardiovascular events (31). A large 5-year randomized controlled trial demonstrated that appropriate atorvastatin use could improve renal function in patients with CAD (32). These findings support the efficacy of antidiabetic and lipid-lowering therapies in preventing AKI among diabetic populations. Our study found that CAD patients with higher TyG levels had an increased risk of AKI, while this risk was reduced in those receiving antidiabetic or lipid-lowering therapies. These results highlight the potential benefit of early metabolic intervention to prevent adverse renal outcomes. In addition to providing valuable guidance for the management of critically ill ICU patients, early pharmacological interventions may help reduce the burden of cardiac and renal complications. These benefits extend to patients with coronary artery disease, both during the acute hospitalization phase and throughout long-term preventive care (33).

Our subgroup analyses further indicated that the modulatory effects of antidiabetic and lipid-lowering therapies may not be consistent across all patient characteristics. For instance, among CAD patients without antidiabetic treatment who had a history of stroke, higher TyG levels were associated with an increased risk of AKI, whereas no such association was observed in patients receiving antidiabetic therapy. Previous studies have indicated that stroke may induce renal dysfunction by altering hemodynamics and neuroendocrine regulation, promoting the release of inflammatory mediators into the circulation and indirectly activating renal inflammatory pathways (34). This finding suggests that achieving adequate glycemic control may provide potential renal protection for CAD patients with a history of stroke. In addition, we explored whether therapeutic approach influenced the association between TyG and AKI. Surprisingly, among patients not receiving antidiabetic or lipid-lowering therapy, higher TyG levels were associated with a lower risk of AKI after CABG. However, this association became nonsignificant in patients treated with these medications. This phenomenon may be explained by the fact that, in the absence of pharmacological intervention, CABG induces a marked systemic inflammatory response and hemodynamic stress, which could mask the chronic metabolic risk reflected by the TyG index. Antidiabetic or lipid-lowering therapies may mitigate surgery-related stress and inflammation, thereby attenuating these differences. Although our study could not stratify precisely by drug type, the consistent reduction in effect sizes across models, along with significant interaction results, suggests a general protective effect of antidiabetic therapy. No significant association between TyG and AKI was observed among patients using lipid-lowering therapy, which may be attributed to both metabolic and non-metabolic effects. Considering the pleiotropic effects of antidiabetic and lipid-lowering drugs, which include enhancing endothelial function, stabilizing atherosclerotic plaques, and reducing systemic inflammation, these drugs may help lower the risk of renal injury independent of their metabolic actions (35). Furthermore, the relatively low number of AKI events in the lipid-lowering group suggests that the nonsignificant findings may partially reflect limited statistical power rather than a true lack of association. Nevertheless, these conclusions should be interpreted with caution given the retrospective design and the lack of detailed information on drug type, dosage, and adherence.

Insulin resistance contributes to renal injury through multiple mechanisms, including endothelial dysfunction, oxidative stress, and inflammatory responses (36, 37). These metabolic abnormalities not only directly damage the kidneys but also exacerbate cardiovascular dysfunction, creating a pathophysiological basis for multi-organ involvement and progression to the cardiorenal-metabolic (CKM) syndrome. CKM is defined as the pathophysiological interplay among high-risk metabolic factors, chronic kidney disease, and the cardiovascular system, ultimately leading to adverse cardiovascular events and organ dysfunction. Therefore, early identification of patients with CVD who have high-risk metabolic factors may effectively prevent or reverse disease progression and reduce the incidence of renal failure (38). In addition to lifestyle modifications to reduce the burden of CKM, pharmacological control of blood glucose and lipids is equally important (39). The use of antidiabetic drugs not only improves renal function but also provides cardioprotective effects, reducing the risk of adverse cardiac and renal events as well as all-cause mortality (40). Furthermore, among patients with CAD, those with metabolic syndrome appear to derive greater renal benefits from lipid-lowering therapy compared with those without metabolic syndrome (41). Invasive interventions such as PCI or CABG may further increase the susceptibility of metabolically high-risk CAD patients to renal injury (42). AKI is a common postoperative complication and is closely associated with progression to end-stage renal disease and increased mortality (43). Therefore, identifying and intervening in metabolically high-risk patients, particularly through pharmacological optimization of glucose and lipid levels, may provide an important strategy to reduce perioperative AKI risk and improve long-term cardiorenal outcomes.

This study has several strengths, including the use of a large and heterogeneous cohort of critically ill patients, the application of multiple statistical approaches, and the use of subgroup and sensitivity analyses to ensure the robustness of the results. In addition, external validation using data from our center further supports the generalizability of the findings. Nevertheless, several limitations should be considered. First, residual confounding from unmeasured variables, such as the etiology and severity of CAD, perioperative management, or metabolic and inflammatory parameters, cannot be excluded. Second, the retrospective design and reliance on database-derived medication data limit the granularity of therapy details, including drug class, dose, duration, and adherence, and the lack of longitudinal follow-up prevents assessment of long-term outcomes. Third, as an observational study, causal inferences cannot be established, and reverse causality remains possible. Fourth, the relatively small number of AKI events in the lipid-lowering drug use subgroup and in the external validation cohort may reduce statistical power to detect significant associations, highlighting the need for future multicenter studies with larger sample sizes. Finally, this study only assessed the relationship between baseline TyG levels and AKI risk in CAD patients under drug use, without accounting for dynamic changes in TyG levels over different treatment durations. Future studies should consider investigating the trajectory of TyG in relation to the duration of therapy.

## Conclusion

In conclusion, this study demonstrates that higher TyG levels are independently associated with an increased risk of AKI in critically ill CAD patients. Notably, antidiabetic and lipid-lowering drug use reduced this risk, suggesting that pharmacological modulation of glucose and lipid metabolism may provide renal protection in this high-risk population. These findings underscore the importance of incorporating metabolic risk assessment into the management of critically ill patients and highlight the potential of drug interventions to improve renal outcomes. Future large-scale prospective studies are needed to confirm causality and explore the underlying mechanisms.

## Data Availability

The datasets presented in this article are not readily available because The data supporting the findings of this study are available from the corresponding author upon reasonable request. Requests to access the datasets should be directed to 15807298713@163.com.
