# Supplementary material for: Antidiabetic and lipid-lowering therapy modify the association between triglyceride–glucose index and acute kidney injury in critically ill patients with coronary artery disease
Source: Front Endocrinol (Lausanne). 2025 Nov 21;16:1699936. doi: 10.3389/fendo.2025.1699936 (PMC12678119; doi:10.3389/fendo.2025.1699936)
Supplement: Supplementary file 1 [file DataSheet1.doc]

**Antidiabetic and lipid-lowering drugs modify the association between TyG index and acute kidney injury in critically ill coronary artery disease patients**

Yuehang Yang^a†^, Linfeng He^b†^, Xueying Jiao^c^, Hanshen Luo^a^, Xiang Qiu^a^, Chuli Shi^a^, Li Ding^a^*^†^, Jiawei Shi^a^*^†^

^a^ Department of Cardiovascular Surgery, Union Hospital, Tongji Medical College, Huazhong University of Science and Technology, Wuhan, Hubei, China

^b^ Department of Endocrinology and Metabolism, The Second Affiliated Hospital, Jiangxi Medical College, Nanchang University, Nanchang, China

^c^ Department of Global Public Health, Karolinska Institutet, Stockholm, Sweden

^†^ These authors contributed equally to this work

*Corresponding author:

Jiawei Shi

Department of Cardiovascular Surgery, Union Hospital, Tongji Medical College, Huazhong University of Science and Technology, 1277 Jiefang Avenue, Wuhan, Hubei, China

1. mail address: 15807298713@163.com

Li Ding

Department of Cardiovascular Surgery, Union Hospital, Tongji Medical College, Huazhong University of Science and Technology, 1277 Jiefang Avenue, Wuhan, Hubei, China

1. mail address: dinglimed@hust.edu.cn

**Supplementary Table S1: Baseline characteristics stratified by antidiabetic drug use.**

| **Variables** | **Overall** | **Non-antidiabetic drug** | **Antidiabetic drug** | ***P* value** |
| --- | --- | --- | --- | --- |
|  | **N = 2,517** | **N = 1,483** | **N = 1,034** |  |
| Age, M (Q₁, Q₃) | 69.00 (61.00 - 78.00) | 70.00 (61.00 - 80.00) | 68.00 (61.00 - 76.00) | 0.002 |
| Male, n (%) | 1,713 (68.06%) | 969 (65.34%) | 744 (71.95%) | <0.001 |
| BMI, M (Q₁, Q₃) | 28.73 (25.02 - 32.91) | 28.06 (24.30 - 32.21) | 29.82 (25.83 - 33.94) | <0.001 |
| Hb, M (Q₁, Q₃) | 10.90 (9.20 - 12.70) | 11.50 (9.60 - 13.10) | 10.20 (8.80 - 11.90) | <0.001 |
| RBC, M (Q₁, Q₃) | 3.66 (3.09 - 4.26) | 3.83 (3.22 - 4.38) | 3.44 (2.94 - 4.06) | <0.001 |
| WBC, M (Q₁, Q₃) | 11.90 (8.60 - 15.90) | 11.30 (8.10 - 15.30) | 12.50 (9.20 - 16.80) | <0.001 |
| PLT, M (Q₁, Q₃) | 188.00 (140.00 - 249.00) | 199.00 (149.00 - 256.00) | 171.00 (128.00 - 236.00) | <0.001 |
| Anion gap, M (Q₁, Q₃) | 14.00 (11.00 - 17.00) | 14.00 (12.00 - 17.00) | 13.00 (10.00 - 16.00) | <0.001 |
| Calcium, M (Q₁, Q₃) | 8.40 (7.90 - 8.80) | 8.40 (7.90 - 8.90) | 8.30 (8.00 - 8.70) | 0.710 |
| Potassium, M (Q₁, Q₃) | 4.30 (3.90 - 4.70) | 4.20 (3.80 - 4.60) | 4.40 (4.00 - 4.80) | <0.001 |
| Sodium, M (Q₁, Q₃) | 138.00 (135.50 - 141.00) | 139.00 (136.00 - 141.00) | 138.00 (135.00 - 140.00) | <0.001 |
| FPG, M (Q₁, Q₃) | 135.00 (110.00 - 179.00) | 129.00 (107.00 - 160.00) | 145.50 (116.00 - 214.00) | <0.001 |
| INR, M (Q₁, Q₃) | 1.30 (1.10 - 1.50) | 1.20 (1.10 - 1.50) | 1.40 (1.20 - 1.60) | 0.443 |
| HDL, M (Q₁, Q₃) | 41.00 (32.00 - 51.00) | 41.50 (32.00 - 52.00) | 40.00 (31.00 - 49.00) | 0.003 |
| LDL, M (Q₁, Q₃) | 76.00 (53.00 - 107.00) | 77.50 (56.00 - 109.00) | 74.00 (51.00 - 104.00) | 0.013 |
| HbA1c, M (Q₁, Q₃) | 5.90 (5.50 - 6.80) | 5.70 (5.40 - 6.00) | 6.40 (5.70 - 8.20) | <0.001 |
| TG, M (Q₁, Q₃) | 119.00 (85.00 - 180.00) | 113.00 (83.00 - 170.00) | 126.50 (88.00 - 192.00) | <0.001 |
| TyG index, M (Q₁, Q₃) | 9.04 (8.60 - 9.58) | 8.95 (8.52 - 9.41) | 9.20 (8.71 - 9.84) | <0.001 |
| ALT, M (Q₁, Q₃) | 34.00 (18.00 - 77.00) | 35.00 (18.00 - 78.00) | 32.00 (18.00 - 76.00) | 0.644 |
| AST, M (Q₁, Q₃) | 54.00 (28.00 - 148.50) | 56.00 (27.00 - 160.00) | 51.00 (29.00 - 139.00) | 0.884 |
| Total bilirubin, M (Q₁, Q₃) | 0.60 (0.40 - 1.00) | 0.60 (0.40 - 1.10) | 0.60 (0.40 - 1.00) | 0.611 |
| Creatinine, M (Q₁, Q₃) | 1.10 (0.80 - 1.60) | 1.10 (0.80 - 1.50) | 1.10 (0.80 - 1.70) | 0.125 |
| Urea nitrogen, M (Q₁, Q₃) | 20.00 (15.00 - 33.00) | 20.00 (15.00 - 31.00) | 20.00 (14.00 - 35.00) | 0.006 |
| SOFA, M (Q₁, Q₃) | 5.00 (3.00 - 8.00) | 4.00 (2.00 - 8.00) | 6.00 (3.00 - 8.00) | <0.001 |
| APSIII, M (Q₁, Q₃) | 43.00 (31.00 - 59.00) | 42.00 (30.00 - 57.00) | 44.00 (32.00 - 62.00) | 0.003 |
| SAPSII, M (Q₁, Q₃) | 37.00 (29.00 - 48.00) | 37.00 (28.00 - 48.00) | 38.00 (31.00 - 49.00) | 0.023 |
| OASIS, M (Q₁, Q₃) | 33.00 (27.00 - 40.00) | 33.00 (27.00 - 40.00) | 33.00 (27.00 - 39.00) | 0.980 |
| Charlson Comorbidity Index, M (Q₁, Q₃) | 6.00 (4.00 - 8.00) | 5.00 (4.00 - 7.00) | 6.00 (4.00 - 8.00) | <0.001 |
| Hypertension, n (%) | 1,081.00 (42.95%) | 659.00 (44.44%) | 422.00 (40.81%) | 0.071 |
| Hepatitis, n (%) | 73.00 (2.90%) | 48.00 (3.24%) | 25.00 (2.42%) | 0.228 |
| Liver cirrhosis, n (%) | 123.00 (4.89%) | 78.00 (5.26%) | 45.00 (4.35%) | 0.299 |
| Stroke, n (%) | 261.00 (10.37%) | 156.00 (10.52%) | 105.00 (10.15%) | 0.768 |
| Cancer, n (%) | 303.00 (12.04%) | 208.00 (14.03%) | 95.00 (9.19%) | <0.001 |
| Hyperlipidemia, n (%) | 1,350 (53.64%) | 711 (47.94%) | 639 (61.80%) | <0.001 |
| COPD, n (%) | 425 (16.89%) | 288 (19.42%) | 137 (13.25%) | <0.001 |
| Diabetes, n (%) | 977 (38.82%) | 309 (20.84%) | 668 (64.60%) | <0.001 |
| Heart failure, n (%) | 1,140 (45.29%) | 656 (44.23%) | 484 (46.81%) | 0.202 |
| CABG, n (%) | 598 (23.76%) | 135 (9.10%) | 463 (44.78%) | <0.001 |
| PCI, n (%) | 405 (16.09%) | 315 (21.24%) | 90 (8.70%) | <0.001 |
| ACEI, n (%) | 1,040 (41.32%) | 652 (43.96%) | 388 (37.52%) | 0.001 |
| ARB, n (%) | 167 (6.63%) | 89 (6.00%) | 78 (7.54%) | 0.126 |
| β-blockers, n (%) | 1,758 (69.85%) | 968 (65.27%) | 790 (76.40%) | <0.001 |

Abbreviations: BMI, body mass index; TyG index, triglyceride-glucose index; Hb, hemoglobin; RBC, red blood cell; WBC, white blood cell; PLT, platelets; FPG, fasting plasma glucose; INR, international normalized ratio; HDL, high density lipoprotein; LDL, lower-density lipoprotein cholesterol; HbA1c, glycosylated hemoglobin, type A1c; TG, triglyceride; TyG, triglyceride-glucose; ALT, alanine aminotransferase; AST, aspartate aminotransferase; SOFA, sequential organ failure assessment; APSIII, acute physiology score; SAPSII, simplified acute physiology score; OASIS, oxford acute severity of illness score; COPD, chronic obstructive pulmonary disease; CABG, coronary artery bypass grafting; PCI, percutaneous coronary intervention; ACEI, angiotensin converting enzyme inhibitors; ARB, angiotensin receptor blocker.

**Supplementary Table S2: Baseline characteristics stratified by lipid lowering drug use.**

| **Variables** | **Overall** | **Non-lipid lowering drug** | **Lipid lowering drug** | ***P* value** |
| --- | --- | --- | --- | --- |
|  | **N = 2,517** | **N = 2,250** | **N = 267** |  |
| Age, M (Q₁, Q₃) | 69.00 (61.00 - 78.00) | 69.00 (61.00 - 78.00) | 72.00 (63.00 - 81.00) | <0.001 |
| Male, n (%) | 1,713 (68.06%) | 1,532 (68.09%) | 181 (67.79%) | 0.921 |
| BMI, M (Q₁, Q₃) | 28.73 (25.02 - 32.91) | 28.77 (24.97 - 32.91) | 28.64 (25.19 - 33.16) | 0.945 |
| Hb, M (Q₁, Q₃) | 10.90 (9.20 - 12.70) | 11.00 (9.30 - 12.80) | 10.50 (8.80 - 12.50) | 0.022 |
| RBC, M (Q₁, Q₃) | 3.66 (3.09 - 4.26) | 3.67 (3.10 - 4.27) | 3.48 (2.95 - 4.15) | 0.046 |
| WBC, M (Q₁, Q₃) | 11.90 (8.60 - 15.90) | 12.00 (8.60 - 16.00) | 11.00 (8.00 - 14.80) | 0.005 |
| PLT, M (Q₁, Q₃) | 188.00 (140.00 - 249.00) | 190.00 (140.00 - 250.00) | 179.00 (134.00 - 243.00) | 0.138 |
| Anion gap, M (Q₁, Q₃) | 14.00 (11.00 - 17.00) | 14.00 (11.00 - 17.00) | 13.00 (11.00 - 16.00) | 0.169 |
| Calcium, M (Q₁, Q₃) | 8.40 (7.90 - 8.80) | 8.40 (7.90 - 8.80) | 8.40 (7.95 - 8.90) | 0.074 |
| Potassium, M (Q₁, Q₃) | 4.30 (3.90 - 4.70) | 4.30 (3.90 - 4.70) | 4.20 (3.90 - 4.70) | 0.926 |
| Sodium, M (Q₁, Q₃) | 138.00 (135.50 - 141.00) | 138.00 (135.00 - 141.00) | 139.00 (136.00 - 140.00) | 0.067 |
| FPG, M (Q₁, Q₃) | 135.00 (110.00 - 179.00) | 136.00 (111.00 - 180.00) | 131.00 (105.00 - 167.00) | 0.110 |
| INR, M (Q₁, Q₃) | 1.30 (1.10 - 1.50) | 1.30 (1.10 - 1.50) | 1.30 (1.20 - 1.50) | 0.399 |
| HDL, M (Q₁, Q₃) | 41.00 (32.00 - 51.00) | 41.00 (32.00 - 51.00) | 38.00 (31.00 - 49.00) | 0.081 |
| LDL, M (Q₁, Q₃) | 76.00 (53.00 - 107.00) | 77.00 (54.00 - 108.00) | 71.00 (49.00 - 99.00) | 0.154 |
| HbA1c, M (Q₁, Q₃) | 5.90 (5.50 - 6.80) | 5.90 (5.50 - 6.80) | 5.90 (5.50 - 6.70) | 0.787 |
| TG, M (Q₁, Q₃) | 119.00 (85.00 - 180.00) | 118.50 (84.00 - 180.00) | 126.00 (91.00 - 190.00) | 0.234 |
| TyG index, M (Q₁, Q₃) | 9.04 (8.60 - 9.58) | 9.04 (8.59 - 9.58) | 9.04 (8.64 - 9.57) | 0.451 |
| ALT, M (Q₁, Q₃) | 34.00 (18.00 - 77.00) | 35.00 (19.00 - 81.00) | 24.00 (15.00 - 53.00) | 0.003 |
| AST, M (Q₁, Q₃) | 54.00 (28.00 - 148.50) | 58.00 (29.00 - 164.00) | 36.00 (23.00 - 83.00) | 0.002 |
| Total bilirubin, M (Q₁, Q₃) | 0.60 (0.40 - 1.00) | 0.60 (0.40 - 1.00) | 0.60 (0.40 - 1.00) | 0.021 |
| Creatinine, M (Q₁, Q₃) | 1.10 (0.80 - 1.60) | 1.10 (0.80 - 1.60) | 1.10 (0.80 - 1.50) | 0.423 |
| Urea nitrogen, M (Q₁, Q₃) | 20.00 (15.00 - 33.00) | 20.00 (15.00 - 33.00) | 20.00 (15.00 - 34.00) | 0.940 |
| SOFA, M (Q₁, Q₃) | 5.00 (3.00 - 8.00) | 5.00 (3.00 - 8.00) | 5.00 (3.00 - 8.00) | 0.224 |
| APSIII, M (Q₁, Q₃) | 43.00 (31.00 - 59.00) | 43.00 (30.00 - 60.00) | 43.00 (33.00 - 57.00) | 0.685 |
| SAPSII, M (Q₁, Q₃) | 37.00 (29.00 - 48.00) | 37.00 (29.00 - 48.00) | 38.00 (31.00 - 49.00) | 0.758 |
| OASIS, M (Q₁, Q₃) | 33.00 (27.00 - 40.00) | 33.00 (27.00 - 40.00) | 32.00 (27.00 - 39.00) | 0.718 |
| Charlson Comorbidity Index, M (Q₁, Q₃) | 6.00 (4.00 - 8.00) | 6.00 (4.00 - 8.00) | 6.00 (4.00 - 8.00) | 0.010 |
| Hypertension, n (%) | 1,081 (42.95%) | 960 (42.67%) | 121 (45.32%) | 0.408 |
| Hepatitis, n (%) | 73 (2.90%) | 67 (2.98%) | 6 (2.25%) | 0.501 |
| Liver cirrhosis, n (%) | 123 (4.89%) | 111 (4.93%) | 12 (4.49%) | 0.753 |
| Stroke, n (%) | 261 (10.37%) | 229 (10.18%) | 32 (11.99%) | 0.360 |
| Cancer, n (%) | 303 (12.04%) | 273 (12.13%) | 30 (11.24%) | 0.670 |
| Hyperlipidemia, n (%) | 1,350 (53.64%) | 1,186 (52.71%) | 164 (61.42%) | 0.007 |
| COPD, n (%) | 425 (16.89%) | 381 (16.93%) | 44 (16.48%) | 0.852 |
| Diabetes, n (%) | 977 (38.82%) | 860 (38.22%) | 117 (43.82%) | 0.076 |
| Heart failure, n (%) | 1,140 (45.29%) | 1,016 (45.16%) | 124 (46.44%) | 0.690 |
| CABG, n (%) | 598 (23.76%) | 515 (22.89%) | 83 (31.09%) | 0.003 |
| PCI, n (%) | 405 (16.09%) | 380 (16.89%) | 25 (9.36%) | 0.002 |
| ACEI, n (%) | 1,040 (41.32%) | 934 (41.51%) | 106 (39.70%) | 0.570 |
| ARB, n (%) | 167 (6.63%) | 144 (6.40%) | 23 (8.61%) | 0.169 |
| β-blockers, n (%) | 1,758 (69.85%) | 1,551 (68.93%) | 207 (77.53%) | 0.004 |

Abbreviations: BMI, body mass index; TyG index, triglyceride-glucose index; Hb, hemoglobin; RBC, red blood cell; WBC, white blood cell; PLT, platelets; FPG, fasting plasma glucose; INR, international normalized ratio; HDL, high density lipoprotein; LDL, lower-density lipoprotein cholesterol; HbA1c, glycosylated hemoglobin, type A1c; TG, triglyceride; TyG, triglyceride-glucose; ALT, alanine aminotransferase; AST, aspartate aminotransferase; SOFA, sequential organ failure assessment; APSIII, acute physiology score; SAPSII, simplified acute physiology score; OASIS, oxford acute severity of illness score; COPD, chronic obstructive pulmonary disease; CABG, coronary artery bypass grafting; PCI, percutaneous coronary intervention; ACEI, angiotensin converting enzyme inhibitors; ARB, angiotensin receptor blocker.

**Supplementary Table S3: Baseline characteristics stratified by AKI.**

| **Variables** | **Overall** | **Non-AKI** | **AKI** | ***P* value** |
| --- | --- | --- | --- | --- |
|  | **N = 2,517** | **N = 586** | **N = 1,931** |  |
| Age, M (Q₁, Q₃) | 69.00 (61.00 - 78.00) | 67.00 (59.00 - 77.00) | 70.00 (62.00 - 79.00) | 0.001 |
| Male, n (%) | 1,713.00 (68.06) | 406.00 (69.28) | 1,307.00 (67.69) | 0.467 |
| BMI, M (Q₁, Q₃) | 28.73 (25.02 - 32.91) | 27.20 (23.91 - 30.86) | 29.26 (25.31 - 33.75) | <0.001 |
| Hb, M (Q₁, Q₃) | 10.90 (9.20 - 12.70) | 11.80 (9.80 - 13.20) | 10.70 (9.10 - 12.50) | <0.001 |
| RBC, M (Q₁, Q₃) | 3.66 (3.09 - 4.26) | 3.91 (3.29 - 4.43) | 3.58 (3.04 - 4.19) | <0.001 |
| WBC, M (Q₁, Q₃) | 11.90 (8.60 - 15.90) | 10.50 (7.80 - 13.60) | 12.31 (8.90 - 16.60) | <0.001 |
| PLT, M (Q₁, Q₃) | 188.00 (140.00 - 249.00) | 200.00 (157.00 - 255.00) | 184.00 (134.00 - 247.50) | 0.001 |
| Anion gap, M (Q₁, Q₃) | 14.00 (11.00 - 17.00) | 13.00 (11.00 - 15.00) | 14.00 (11.00 - 17.00) | <0.001 |
| Calcium, M (Q₁, Q₃) | 8.40 (7.90 - 8.80) | 8.60 (8.10 - 9.00) | 8.30 (7.90 - 8.70) | <0.001 |
| Potassium, M (Q₁, Q₃) | 4.30 (3.90 - 4.70) | 4.20 (3.90 - 4.50) | 4.30 (3.90 - 4.70) | <0.001 |
| Sodium, M (Q₁, Q₃) | 138.00 (135.50 - 141.00) | 138.00 (136.00 - 140.00) | 138.00 (135.00 - 141.00) | 0.893 |
| FPG, M (Q₁, Q₃) | 135.00 (110.00 - 179.00) | 122.50 (104.00 - 150.00) | 139.00 (113.00 - 187.00) | <0.001 |
| INR, M (Q₁, Q₃) | 1.30 (1.10 - 1.50) | 1.20 (1.10 - 1.40) | 1.30 (1.20 - 1.60) | <0.001 |
| HDL, M (Q₁, Q₃) | 41.00 (32.00 - 51.00) | 44.00 (35.00 - 53.00) | 39.00 (31.00 - 50.00) | <0.001 |
| LDL, M (Q₁, Q₃) | 76.00 (53.00 - 107.00) | 85.00 (61.00 - 115.00) | 73.00 (51.00 - 103.00) | <0.001 |
| HbA1c, M (Q₁, Q₃) | 5.90 (5.50 - 6.80) | 5.80 (5.50 - 6.50) | 5.90 (5.50 - 6.90) | 0.451 |
| TG, M (Q₁, Q₃) | 119.00 (85.00 - 180.00) | 106.00 (76.00 - 150.00) | 124.00 (87.00 - 191.00) | <0.001 |
| TyG index, M (Q₁, Q₃) | 9.04 (8.60 - 9.58) | 8.84 (8.39 - 9.25) | 9.09 (8.65 - 9.70) | <0.001 |
| ALT, M (Q₁, Q₃) | 34.00 (18.00 - 77.00) | 27.00 (17.00 - 51.00) | 35.00 (18.00 - 87.00) | <0.001 |
| AST, M (Q₁, Q₃) | 54.00 (28.00 - 148.50) | 44.00 (24.00 - 105.50) | 57.00 (30.00 - 160.50) | <0.001 |
| Total bilirubin, M (Q₁, Q₃) | 0.60 (0.40 - 1.00) | 0.50 (0.40 - 0.80) | 0.70 (0.40 - 1.10) | <0.001 |
| Creatinine, M (Q₁, Q₃) | 1.10 (0.80 - 1.60) | 0.90 (0.70 - 1.20) | 1.10 (0.90 - 1.80) | <0.001 |
| Urea nitrogen, M (Q₁, Q₃) | 20.00 (15.00 - 33.00) | 17.00 (13.00 - 22.00) | 22.00 (15.00 - 36.00) | <0.001 |
| SOFA, M (Q₁, Q₃) | 5.00 (3.00 - 8.00) | 2.00 (1.00 - 4.00) | 6.00 (4.00 - 9.00) | <0.001 |
| APSIII, M (Q₁, Q₃) | 43.00 (31.00 - 59.00) | 30.00 (24.00 - 41.00) | 48.00 (34.00 - 66.00) | <0.001 |
| SAPSII, M (Q₁, Q₃) | 37.00 (29.00 - 48.00) | 29.00 (23.00 - 36.00) | 40.00 (32.00 - 51.00) | <0.001 |
| OASIS, M (Q₁, Q₃) | 33.00 (27.00 - 40.00) | 27.00 (22.00 - 32.00) | 35.00 (29.00 - 41.00) | <0.001 |
| Charlson Comorbidity Index, M (Q₁, Q₃) | 6.00 (4.00 - 8.00) | 5.00 (3.00 - 7.00) | 6.00 (4.00 - 8.00) | <0.001 |
| Hypertension, n (%) | 1,081.00 (42.95) | 288.00 (49.15) | 793.00 (41.07) | <0.001 |
| Hepatitis, n (%) | 73.00 (2.90) | 6.00 (1.02) | 67.00 (3.47) | 0.002 |
| Liver cirrhosis, n (%) | 123.00 (4.89) | 6.00 (1.02) | 117.00 (6.06) | <0.001 |
| Stroke, n (%) | 261.00 (10.37) | 51.00 (8.70) | 210.00 (10.88) | 0.131 |
| Cancer, n (%) | 303.00 (12.04) | 77.00 (13.14) | 226.00 (11.70) | 0.349 |
| Hyperlipidemia, n (%) | 1,350.00 (53.64) | 354.00 (60.41) | 996.00 (51.58) | <0.001 |
| COPD, n (%) | 425.00 (16.89) | 77.00 (13.14) | 348.00 (18.02) | 0.006 |
| Diabetes, n (%) | 977.00 (38.82) | 180.00 (30.72) | 797.00 (41.27) | <0.001 |
| Heart failure, n (%) | 1,140.00 (45.29) | 191.00 (32.59) | 949.00 (49.15) | <0.001 |
| CABG, n (%) | 598.00 (23.76) | 134.00 (22.87) | 464.00 (24.03) | 0.563 |
| PCI, n (%) | 405.00 (16.09) | 176.00 (30.03) | 229.00 (11.86) | <0.001 |
| ACEI, n (%) | 1,040.00 (41.32) | 306.00 (52.22) | 734.00 (38.01) | <0.001 |
| ARB, n (%) | 167.00 (6.63) | 34.00 (5.80) | 133.00 (6.89) | 0.355 |
| β-blockers, n (%) | 1,758.00 (69.85) | 456.00 (77.82) | 1,302.00 (67.43) | <0.001 |

Abbreviations: AKI, acute kidney injury; BMI, body mass index; TyG index, triglyceride-glucose index; Hb, hemoglobin; RBC, red blood cell; WBC, white blood cell; PLT, platelets; FPG, fasting plasma glucose; INR, international normalized ratio; HDL, high density lipoprotein; LDL, lower-density lipoprotein cholesterol; HbA1c, glycosylated hemoglobin, type A1c; TG, triglyceride; TyG, triglyceride-glucose; ALT, alanine aminotransferase; AST, aspartate aminotransferase; SOFA, sequential organ failure assessment; APSIII, acute physiology score; SAPSII, simplified acute physiology score; OASIS, oxford acute severity of illness score; COPD, chronic obstructive pulmonary disease; CABG, coronary artery bypass grafting; PCI, percutaneous coronary intervention; ACEI, angiotensin converting enzyme inhibitors; ARB, angiotensin receptor blocker.

**Supplementary Table S4: Baseline characteristics stratified by antidiabetic drug use in the external validation data.**

| **Variables** | **Overall**  **N = 910** | **Non-antidiabetic drug**  **N =764** | **Antidiabetic drug**  **N = 146** | ***P* value** |
| --- | --- | --- | --- | --- |
|
| Age, M (Q₁, Q₃) | 59.00 (53.00, 65.00) | 59.00 (53.00, 64.00) | 59.00 (54.00, 65.00) | 0.299 |
| Male, n(%) | 557 (61.21) | 457 (59.82) | 100 (68.49) | 0.049 |
| BMI, M (Q₁, Q₃) | 23.51 (21.60, 25.77) | 23.40 (21.48, 25.71) | 24.43 (22.04, 26.36) | 0.005 |
| Smoking, n(%) | 231 (25.38) | 185 (24.21) | 46 (31.51) | 0.064 |
| Drinking, n(%) | 181 (19.89) | 140 (18.32) | 41 (28.08) | 0.007 |
| Hb, M (Q₁, Q₃) | 129.00 (118.00, 139.00) | 128.00 (118.00, 139.00) | 131.00 (119.00, 140.75) | 0.232 |
| PT, M (Q₁, Q₃) | 13.50 (13.00, 14.38) | 13.50 (13.00, 14.20) | 13.70 (13.03, 14.70) | 0.005 |
| ALT, M (Q₁, Q₃) | 20.00 (15.00, 29.00) | 20.00 (15.00, 29.00) | 22.00 (16.00, 29.75) | 0.071 |
| AST, M (Q₁, Q₃) | 23.00 (19.00, 29.00) | 23.00 (19.00, 29.00) | 22.50 (18.00, 28.00) | 0.583 |
| LDL, M (Q₁, Q₃) | 2.36 (2.00, 2.67) | 2.36 (2.04, 2.67) | 2.36 (1.85, 2.68) | 0.197 |
| Albumin, M (Q₁, Q₃) | 39.08 (36.50, 41.30) | 39.20 (36.77, 41.30) | 38.50 (35.65, 41.00) | 0.047 |
| Creatinine, M (Q₁, Q₃) | 74.00 (63.10, 86.30) | 73.35 (62.30, 85.53) | 79.90 (67.03, 92.83) | 0.001 |
| Urea nitrogen, M (Q₁, Q₃) | 6.20 (5.22, 7.57) | 6.17 (5.22, 7.41) | 6.54 (5.40, 8.33) | 0.013 |
| TG, M (Q₁, Q₃) | 117.50 (86.00, 176.00) | 113.00 (84.00, 171.25) | 132.50 (102.00, 201.50) | <0.001 |
| FPG, M (Q₁, Q₃) | 133.00 (108.00, 171.00) | 131.00 (108.00, 163.00) | 147.00 (116.25, 229.25) | <0.001 |
| TyG index, M (Q₁, Q₃) | 9.04 (8.61, 9.55) | 8.96 (8.57, 9.49) | 9.41 (8.91, 9.84) | <0.001 |
| NT-proBNP, M (Q₁, Q₃) | 299.00 (67.28, 839.50) | 268.50 (64.30, 705.75) | 483.50 (116.25, 1527.50) | <0.001 |
| Myocardial infarction, n(%) | 2 (0.22) | 1 (0.13) | 1 (0.68) | 0.295 |
| Coronary stent implantation, n(%) | 9 (0.99) | 5 (0.65) | 4 (2.74) | 0.061 |
| Hypertension, n(%) | 308 (33.85) | 239 (31.28) | 69 (47.26) | <0.001 |
| Diabetes, n(%) | 120 (13.19) | 21 (2.75) | 99 (67.81) | <0.001 |
| COPD, n(%) | 123 (13.52) | 97 (12.70) | 26 (17.81) | 0.098 |
| Stroke, n(%) | 253 (27.80) | 199 (26.05) | 54 (36.99) | 0.007 |
| β-blockers, n(%) | 389 (42.75) | 290 (37.96) | 99 (67.81) | <0.001 |
| ACEI/ARB, n(%) | 237 (26.04) | 145 (18.98) | 92 (63.01) | <0.001 |
| Aspirin, n(%) | 171 (18.79) | 135 (17.67) | 36 (24.66) | 0.048 |
| Clopidogrel, n(%) | 56 (6.15) | 47 (6.15) | 9 (6.16) | 0.995 |
| Length of stay, M (Q₁, Q₃) | 23.00 (19.00, 30.00) | 22.00 (18.00, 29.00) | 26.00 (21.00, 33.00) | | <0.001 | | --- | |
| Duration of ventilator use (h), M (Q₁, Q₃) | 19.00 (14.00, 21.00) | 18.00 (14.00, 21.00) | 20.00 (17.00, 25.00) | <0.001 |
| Length of stay in the ICU (day), M (Q₁, Q₃) | 2.00 (2.00, 3.00) | 2.00 (2.00, 3.00) | 3.00 (2.00, 4.00) | 0.021 |

Abbreviations: BMI, body mass index; Hb, hemoglobin; PT, prothrombin time; ALT, alanine aminotransferase; AST, aspartate aminotransferase; LDL, lower-density lipoprotein cholesterol; TG, triglyceride; FPG, fasting plasma glucose; TyG, triglyceride-glucose; NT-proBNP, N-terminal pro-brain natriuretic peptide; COPD, chronic obstructive pulmonary disease; ACEI, angiotensin converting enzyme inhibitors; ARB, angiotensin receptor blocker.

**Supplementary Table S5: Baseline characteristics stratified by lipid lowering drug use in the external validation data.**

| **Variables** | **Overall**  **N = 910** | **Non-lipid lowering drug**  **N = 693** | **Lipid lowering drug**  **N = 217** | ***P* value** |
| --- | --- | --- | --- | --- |
|
| Age, M (Q₁, Q₃) | 59.00 (53.00, 65.00) | 58.00 (53.00, 64.00) | 60.00 (54.00, 66.00) | 0.009 |
| Male, n(%) | 557 (61.21) | 401 (57.86) | 156 (71.89) | <0.001 |
| BMI, M (Q₁, Q₃) | 23.51 (21.60, 25.77) | 23.34 (21.56, 25.65) | 24.22 (21.78, 26.42) | 0.026 |
| Smoking, n(%) | 231 (25.38) | 152 (21.93) | 79 (36.41) | <0.001 |
| Drinking, n(%) | 181 (19.89) | 118 (17.03) | 63 (29.03) | <0.001 |
| Hb, M (Q₁, Q₃) | 129.00 (118.00, 139.00) | 128.00 (117.00, 139.00) | 131.00 (122.00, 141.00) | 0.003 |
| PT, M (Q₁, Q₃) | 13.50 (13.00, 14.38) | 13.50 (13.00, 14.30) | 13.60 (13.00, 14.38) | 0.562 |
| ALT, M (Q₁, Q₃) | 20.00 (15.00, 29.00) | 19.00 (14.00, 28.00) | 24.00 (17.00, 32.00) | <0.001 |
| AST, M (Q₁, Q₃) | 23.00 (19.00, 29.00) | 22.00 (19.00, 28.00) | 24.00 (20.00, 30.00) | 0.033 |
| LDL, M (Q₁, Q₃) | 2.36 (2.00, 2.67) | 2.36 (2.11, 2.70) | 2.36 (1.69, 2.54) | <0.001 |
| Albumin, M (Q₁, Q₃) | 39.08 (36.50, 41.30) | 39.10 (36.70, 41.20) | 39.08 (36.30, 41.30) | 0.894 |
| Creatinine, M (Q₁, Q₃) | 74.00 (63.10, 86.30) | 73.20 (61.90, 85.10) | 78.30 (66.50, 94.10) | <0.001 |
| Urea nitrogen, M (Q₁, Q₃) | 6.20 (5.22, 7.57) | 6.16 (5.21, 7.47) | 6.38 (5.27, 7.96) | 0.143 |
| TG, M (Q₁, Q₃) | 117.50 (86.00, 176.00) | 112.00 (84.00, 166.00) | 138.00 (96.00, 208.00) | <0.001 |
| FPG, M (Q₁, Q₃) | 133.00 (108.00, 171.00) | 132.00 (108.00, 170.00) | 136.00 (109.00, 174.00) | 0.581 |
| TyG index, M (Q₁, Q₃) | 9.04 (8.61, 9.55) | 8.98 (8.58, 9.49) | 9.21 (8.76, 9.72) | <0.001 |
| NT-proBNP, M (Q₁, Q₃) | 299.00 (67.28, 839.50) | 260.00 (64.30, 712.00) | 393.00 (112.00, 1010.00) | 0.001 |
| Myocardial infarction, n(%) | 2 (0.22) | 1 (0.14) | 1 (0.46) | 0.420 |
| Coronary stent implantation, n(%) | 9 (0.99) | 3 (0.43) | 6 (2.76) | 0.008 |
| Hypertension, n(%) | 308 (33.85) | 207 (29.87) | 101 (46.54) | <0.001 |
| Diabetes, n(%) | 120 (13.19) | 75 (10.82) | 45 (20.74) | <0.001 |
| COPD, n(%) | 123 (13.52) | 97 (14.00) | 26 (11.98) | 0.449 |
| Stroke, n(%) | 253 (27.80) | 181 (26.12) | 72 (33.18) | 0.043 |
| β-blockers, n(%) | 389 (42.75) | 246 (35.50) | 143 (65.90) | <0.001 |
| ACEI/ARB, n(%) | 237 (26.04) | 140 (20.20) | 97 (44.70) | <0.001 |
| Aspirin, n(%) | 171 (18.79) | 90 (12.99) | 81 (37.33) | <0.001 |
| Clopidogrel, n(%) | 56 (6.15) | 28 (4.04) | 28 (12.90) | <0.001 |
| Length of stay, M (Q₁, Q₃) | 23.00 (19.00, 30.00) | 23.00 (19.00, 29.00) | 24.00 (19.00, 32.00) | 0.035 |
| Duration of ventilator use (h), M (Q₁, Q₃) | 19.00 (14.00, 21.00) | 19.00 (14.00, 21.00) | 19.00 (14.00, 22.00) | 0.438 |
| Length of stay in the ICU (day), M (Q₁, Q₃) | 2.00 (2.00, 3.00) | 2.00 (2.00, 3.00) | 2.00 (2.00, 4.00) | 0.155 |

Abbreviations: BMI, body mass index; Hb, hemoglobin; PT, prothrombin time; ALT, alanine aminotransferase; AST, aspartate aminotransferase; LDL, lower-density lipoprotein cholesterol; TG, triglyceride; FPG, fasting plasma glucose; TyG, triglyceride-glucose; NT-proBNP, N-terminal pro-brain natriuretic peptide; COPD, chronic obstructive pulmonary disease; ACEI, angiotensin converting enzyme inhibitors; ARB, angiotensin receptor blocker.

**Supplementary Table S6: Baseline characteristics stratified by AKI in the external validation data.**

| **Variables** | **Overall**  **N =910** | **Non-AKI**  **N = 781** | **AKI**  **N = 129** | **P value** |
| --- | --- | --- | --- | --- |
|
| Age, M (Q₁, Q₃) | 59.00 (53.00, 65.00) | 59.00 (53.00, 64.00) | 60.00 (54.00, 65.00) | 0.111 |
| Male, n (%) | 557 (61.21) | 485 (62.10) | 72 (55.81) | 0.175 |
| BMI, M (Q₁, Q₃) | 23.51 (21.60, 25.77) | 23.46 (21.55, 25.71) | 23.61 (22.06, 26.22) | 0.349 |
| Smoking, n(%) | 231 (25.38) | 197 (25.22) | 34 (26.36) | 0.784 |
| Drinking, n(%) | 181 (19.89) | 154 (19.72) | 27 (20.93) | 0.749 |
| Hb, M (Q₁, Q₃) | 129.00 (118.00, 139.00) | 129.00 (119.00, 139.00) | 128.00 (117.00, 139.00) | 0.309 |
| PT, M (Q₁, Q₃) | 13.50 (13.00, 14.38) | 13.50 (13.00, 14.38) | 13.50 (13.00, 14.20) | 0.753 |
| ALT, M (Q₁, Q₃) | 20.00 (15.00, 29.00) | 20.00 (15.00, 29.00) | 21.00 (15.00, 33.00) | 0.231 |
| AST, M (Q₁, Q₃) | 23.00 (19.00, 29.00) | 23.00 (19.00, 28.00) | 23.00 (20.00, 30.00) | 0.186 |
| LDL, M (Q₁, Q₃) | 2.36 (2.00, 2.67) | 2.36 (2.00, 2.66) | 2.36 (2.01, 2.75) | 0.744 |
| Albumin, M (Q₁, Q₃) | 39.08 (36.50, 41.30) | 39.20 (36.50, 41.30) | 38.60 (36.20, 41.00) | 0.160 |
| Creatinine, M (Q₁, Q₃) | 74.00 (63.10, 86.30) | 76.20 (63.70, 88.00) | 67.30 (57.60, 78.80) | <0.001 |
| Urea nitrogen, M (Q₁, Q₃) | 6.20 (5.22, 7.57) | 6.26 (5.25, 7.58) | 5.75 (4.98, 7.39) | 0.060 |
| TG, M (Q₁, Q₃) | 117.50 (86.00, 176.00) | 115.00 (85.00, 172.00) | 134.00 (98.00, 221.00) | 0.003 |
| FPG, M (Q₁, Q₃) | 133.00 (108.00, 171.00) | 131.00 (108.00, 167.00) | 140.00 (108.00, 206.00) | 0.008 |
| TyG index, M (Q₁, Q₃) | 9.04 (8.61, 9.55) | 8.98 (8.58, 9.50) | 9.35 (8.80, 10.10) | <0.001 |
| NT-proBNP, M (Q₁, Q₃) | 299.00 (67.28, 839.50) | 285.00 (64.70, 809.00) | 389.00 (85.20, 957.00) | 0.130 |
| Myocardial infarction, n(%) | 2 (0.22) | 1 (0.13) | 1 (0.78) | 0.264 |
| Coronary stent implantation, n(%) | 9 (0.99) | 7 (0.90) | 2 (1.55) | 0.830 |
| Hypertension, n(%) | 308 (33.85) | 259 (33.16) | 49 (37.98) | 0.284 |
| Diabetes, n(%) | 119 (13.19) | 90 (11.65) | 29 (22.48) | 0.002 |
| COPD, n(%) | 123 (13.52) | 106 (13.57) | 17 (13.18) | 0.903 |
| Stroke, n(%) | 253 (27.80) | 229 (29.32) | 24 (18.60) | 0.012 |
| β-blockers, n (%) | 389 (42.75) | 325 (41.61) | 64 (49.61) | 0.089 |
| ACEI/ARB, n(%) | 237 (26.04) | 192 (24.58) | 45 (34.88) | 0.014 |
| Aspirin, n(%) | 171 (18.79) | 147 (18.82) | 24 (18.60) | 0.953 |
| Clopidogrel, n(%) | 56 (6.15) | 44 (5.63) | 12 (9.30) | 0.108 |
| Length of stay, M (Q₁, Q₃) | 23.00 (19.00, 30.00) | 23.00 (19.00, 29.00) | 26.00 (21.00, 32.00) | 0.003 |
| Duration of ventilator use (h), M (Q₁, Q₃) | 19.00 (14.00, 21.00) | 19.00 (14.00, 21.00) | 19.00 (15.00, 22.00) | 0.303 |
| Length of stay in the ICU (day), M (Q₁, Q₃) | 2.00 (2.00, 3.00) | 2.00 (2.00, 3.00) | 2.00 (2.00, 4.00) | 0.058 |

Abbreviations: BMI, body mass index; Hb, hemoglobin; PT, prothrombin time; ALT, alanine aminotransferase; AST, aspartate aminotransferase; LDL, lower-density lipoprotein cholesterol; TG, triglyceride; FPG, fasting plasma glucose; TyG, triglyceride-glucose; NT-proBNP, N-terminal pro-brain natriuretic peptide; COPD, chronic obstructive pulmonary disease; ACEI, angiotensin converting enzyme inhibitors; ARB, angiotensin receptor blocker.

**Supplementary Table S7: Association between the TyG index and AKI Stratified by antidiabetic drug use in the external validation data.**

|  | | **Number of incidence (rate)** | | **Model I**  **OR (95%CI)** | ***P*** | **Model II**  **OR (95%CI)** | ***P*** | **Model III**  **OR (95%CI)** | ***P*** | ***P* for interaction** | |
| --- | --- | --- | --- | --- | --- | --- | --- | --- | --- | --- | --- |
| **Without antidiabetic drug** 0.276 | | | | | | | | | | | |
| Continuous variable per 1 unit 1.880(1.520,2.340) <0.001 1.884(1.522,2.345) <0.001 1.912(1.540,2.387) <0.001  **Tertile** | | | | | | | | | | | |
| TyG index | | | | | | | | | | | |
| T1 (≤8.76) | | 57(22.44) | | Ref |  | Ref |  | Ref |  |  | |
| T2 (8.76-9.38) | | 71(27.95) | | 1.341(0.562,1.780) | 0.153 | 1.327(0.888,1.991) | 0.169 | 1.365(0.910,2.056) | 0.134 |
| T3 (≥9.38) | | 116(45.31) | | 2.864(1.958,4.224) | <0.001 | 2.860(1.954,4.222) | <0.001 | 2.946(2.002,4.373) | <0.001 |
| P for trend | |  |  |  | 0.005 |  | 0.005 |  | 0.004 |  |  |
| **With antidiabetic drug**  Continuous variable per 1 unit 1.532(0.892,2.685) 0.126 1.435(0.815,2.595) 0.214 1.466(0.817,2.722) 0.209  **Tertile** | | | | | | | | | | | |
| TyG index |  | | | | | | | | | | |
| T1 (≤8.76) | | 11(22.92) | | Ref |  | Ref |  | Ref |  |  | |
| T2 (8.76-9.38) | | 10(20.83) | | 1.267(0.487,3.350) | 0.628 | 1.156(0.426,3.178) | 0.775 | 1.168(0.415,3.312) | 0.767 |
| T3 (≥9.38) | | 13(26.00) | | 1.629(0.653,4.198) | 0.300 | 1.432(0.546,3.855) | 0.467 | 1.540(0.570,4.275) | 0.397 |
| P for trend | |  | |  | 0.879 |  | 0.985 |  | 0.825 |  | |

Note: Model I: Unadjusted

Model II: Adjusted for age, sex, BMI

Model III: Adjusted for age, sex, BMI, COPD, hypertension, stroke

Abbreviations: 95% CI: 95% confidence interval; OR: odds ratio; Ref: reference; TyG: triglyceride-glucose

**P* for interactions was calculated for Model III

**Supplementary Table S8: Association between the TyG index and AKI stratified by lipid-lowering drug use in the external validation data.**

|  | | **Number of incidence (rate)** | | **Model I**  **OR (95%CI)** | ***P*** | **Model II**  **OR (95%CI)** | ***P*** | **Model III**  **OR (95%CI)** | ***P*** | ***P* for interaction** | |
| --- | --- | --- | --- | --- | --- | --- | --- | --- | --- | --- | --- |
| **Without lipid-lowering drug** 0.055  Continuous variable per 1 unit 1.693(1.349,2.134) <0.001 1.678(1.344,2.129) <0.001 1.716(1.363,2.171) <0.001  **Tertile** | | | | | | | | | | | |
| TyG index | | | | | | | | | | | |
| T1 (≤8.76) | | 52(22.51) | | Ref |  | Ref |  | Ref |  |  | |
| T2 (8.76-9.38) | | 63(27.27) | | 1.731(1.146,2.630) | 0.010 | 1.709(1.130,2.600) | 0.012 | 1.734(1.142,2.649) | 0.010 |
| T3 (≥9.38) | | 89(38.53) | | 2.379(1.589,3.592) | <0.001 | 2.335(1.557,3.531) | <0.001 | 2.411(1.602,3.663) | <0.001 |
| P for trend | |  |  |  | <0.001 |  | <0.001 |  | <0.001 |  |  |
| **With lipid-lowering drug**  Continuous variable per 1 unit 1.108(0.763,1.617) 0.590 1.104(0.760,1.612) 0.603 1.116(0.766,1.636) 0.567  **Tertile** | | | | | | | | | | | |
| TyG index |  | | | | | | | | | | |
| T1 (≤8.76) | | 15(20.83) | | Ref |  | Ref |  | Ref |  |  | |
| T2 (8.76-9.38) | | 19(26.39) | | 1.362(0.630,2.987) | 0.433 | 1.379(0.629,3.068) | 0.424 | 1.383(0.621,3.133) | 0.429 |
| T3 (≥9.38) | | 22(30.14) | | 1.639(0.774,3.548) | 0.201 | 1.612(0.755,3.516) | 0.222 | 1.742(0.804,3.869) | 0.164 |
| P for trend | |  | |  | 0.202 |  | 0.224 |  | 0.164 |  | |

Note: Model I: Unadjusted

Model II: Adjusted for age, sex, BMI

Model III: Adjusted for age, sex, BMI, COPD, hypertension, stroke

Abbreviations: 95% CI: 95% confidence interval; OR: odds ratio; Ref: reference; TyG: triglyceride-glucose

**P* for interactions was calculated for Model III

Supplementary Table S9: Association between the TyG index and AKI after inclusion of individuals previously excluded due to missing BMI data: Stratified by antidiabetic drug use.

|  | | **Number of incidence (rate)** | | **Model I**  **OR (95%CI)** | ***P*** | **Model II**  **OR (95%CI)** | ***P*** | **Model III**  **OR (95%CI)** | ***P*** | ***P* for interaction** | |
| --- | --- | --- | --- | --- | --- | --- | --- | --- | --- | --- | --- |
| **Without antidiabetic drug** 0.004 | | | | | | | | | | | |
| Continuous variable per 1 unit 1.659(1.471,1.876) <0.001 1.762(1.552,2.001) <0.001 1.808(1.592,2.061) <0.001  **Tertile** | | | | | | | | | | | |
| TyG index | | | | | | | | | | | |
| T1 (≤8.70) | | 474(54.23) | | Ref |  | Ref |  | Ref |  |  | |
| T2 (8.70-9.30) | | 546(62.47) | | 1.405(1.161,1.701) | <0.001 | 1.455(1.201,1.764) | <0.001 | 1.489(1.224,1.813) | <0.001 |
| T3 (≥9.30) | | 623(71.28) | | 2.095(1.720,2.555) | <0.001 | 2.293(1.873,2.812) | <0.001 | 2.371(1.929,2.920) | <0.001 |
| P for trend | |  |  |  | <0.001 |  | <0.001 |  | <0.001 |  |  |
| **With antidiabetic drug**  Continuous variable per 1 unit 1.300(1.121,1.512) <0.001 1.357(1.166,1.583) <0.001 1.349(1.158,1.577) <0.001  **Tertile** | | | | | | | | | | | |
| TyG index |  | | | | | | | | | | |
| T1 (≤8.70) | | 338(71.16) | | Ref |  | Ref |  | Ref |  |  | |
| T2 (8.70-9.30) | | 354(74.53) | | 1.186(0.891,1.580) | 0.243 | 1.211(0.908,1.616) | 0.193 | 1.227(0.919,1.640) | 0.167 |
| T3 (≥9.30) | | 382(80.25) | | 1.647(1.221,2.229) | 0.001 | 1.771(1.307,2.411) | <0.001 | 1.741(1.280,2.375) | <0.001 |
| P for trend | |  | |  | 0.001 |  | <0.001 |  | <0.001 |  | |

Note: Model I: Unadjusted

Model II: Adjusted for age, sex

Model III: Adjusted for age, sex, COPD, hypertension, liver cirrhosis, stroke, hyperlipemia

Abbreviations: 95% CI: 95% confidence interval; OR: odds ratio; Ref: reference; TyG: triglyceride-glucose

**P* for interactions was calculated for Model III

Supplementary Table S10: Association between the TyG index and AKI after inclusion of individuals previously excluded due to missing BMI data: Stratified by lipid-lowering drug use.

|  | | **Number of incidence (rate)** | | **Model I**  **OR (95%CI)** | ***P*** | **Model II**  **OR (95%CI)** | ***P*** | **Model III**  **OR (95%CI)** | ***P*** | ***P* for interaction** | |
| --- | --- | --- | --- | --- | --- | --- | --- | --- | --- | --- | --- |
| **Without lipid-lowering drug** 0.008 | | | | | | | | | | | |
| Continuous variable per 1 unit 1.673(1.517,1.847) <0.001 1.771(1.602,1.962) <0.001 1.806(1.630,2.004) <0.001  **Tertile** | | | | | | | | | | | |
| TyG index | | | | | | | | | | | |
| T1 (≤8.70) | | 705(58.70) | | Ref |  | Ref |  | Ref |  |  | |
| T2 (8.70-9.30) | | 793(66.03) | | 1.367(1.159,1.614) | <0.001 | 1.411(1.195,1.668) | <0.001 | 1.453(1.227,1.721) | <0.001 |
| T3 (≥9.30) | | 911(75.85) | | 2.210(1.856,2.635) | <0.001 | 2.443(2.042,2.929) | <0.001 | 2.506(2.089,3.011) | <0.001 |
| P for trend | |  |  |  | <0.001 |  | <0.001 |  | <0.001 |  |  |
| **With lipid-lowering drug**  Continuous variable per 1 unit 1.169(0.911,1.517) 0.227 1.166(0.905,1.519) 0.242 1.147(0.889,1.497) 0.299  **Tertile** | | | | | | | | | | | |
| TyG index |  | | | | | | | | | | |
| T1 (≤8.70) | | 93(62.84) | | Ref |  | Ref |  | Ref |  |  | |
| T2 (8.70-9.30) | | 105(70.95) | | 1.444(0.889,2.358) | 0.139 | 1.455(0.890,2.392) | 0.136 | 1.418(0.861,2.347) | 0.171 |
| T3 (≥9.30) | | 110(73.83) | | 1.668(1.020,2.748) | 0.043 | 1.685(1.018,2.809) | 0.044 | 1.656(0.994,2.778) | 0.054 |
| P for trend | |  | |  | 0.041 |  | 0.043 |  | 0.053 |  | |

Note: Model I: Unadjusted

Model II: Adjusted for age, sex

Model III: Adjusted for age, sex, COPD, hypertension, liver cirrhosis, stroke, hyperlipemia

Abbreviations: 95% CI: 95% confidence interval; OR: odds ratio; Ref: reference; TyG: triglyceride-glucose

**P* for interactions was calculated for Model III


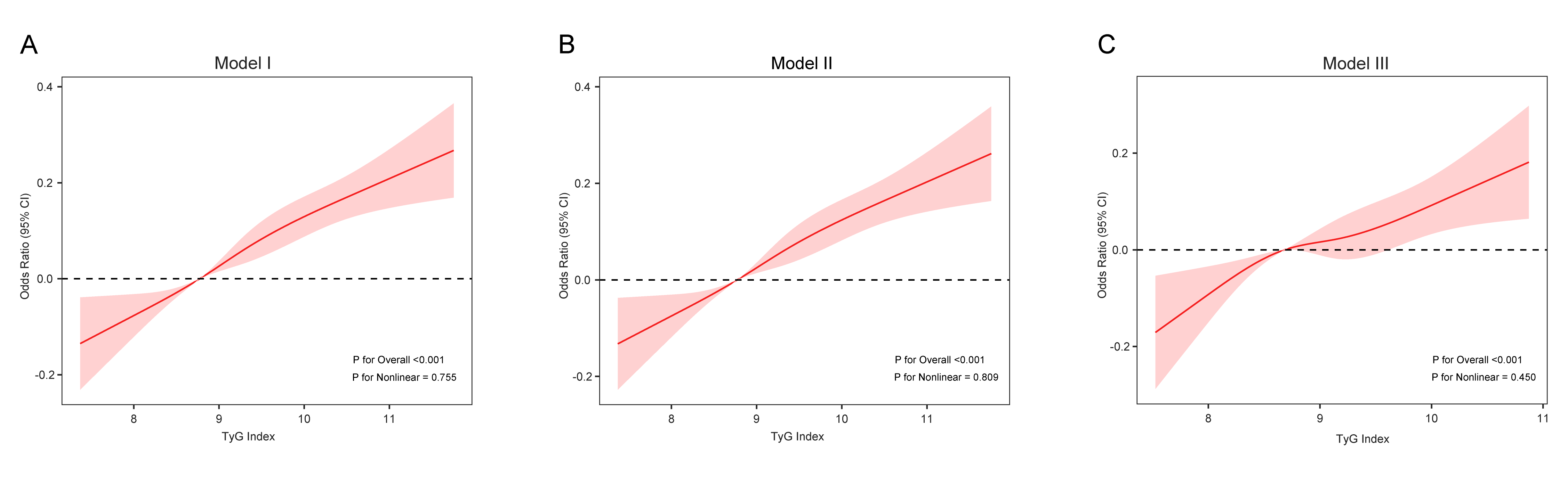
**Supplementary Figure S1:** **Association of TyG index with AKI**. (A) Model I: unadjusted; (B) Model II:adjusted for age, sex, and BMI; (C) Model III:adjusted for age, sex, BMI, hypertension, stroke, hyperlipidemia, liver cirrhosis and COPD. Abbreviations: TyG index, triglyceride-glucose index.


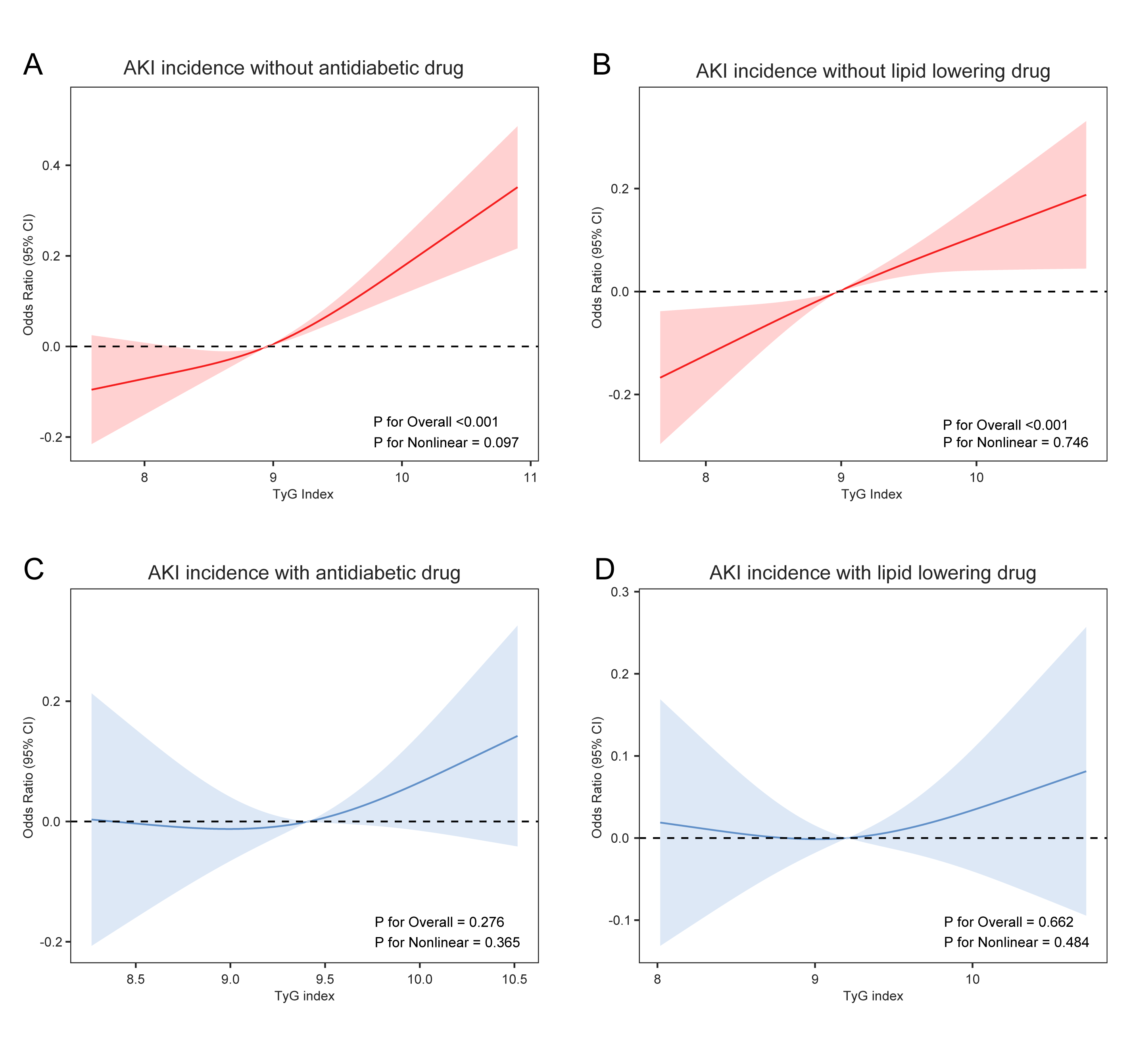
**Supplementary Figure S2:** **In the external validation data, association of the TyG index with AKI, stratified by antidiabetic and lipid-lowering drug use.** (A) Restricted cubic spline curve for the AKI without antidiabetic drug; (B) Restricted cubic spline curve for the AKI without lipid lowering drug; (C) Restricted cubic spline curve for the AKI with antidiabetic drug; (D) Restricted cubic spline curve for the AKI with lipid lowering drug. The non-medication group was indicated in red, while the medication group was indicated in blue, shaded areas indicate 95% CIs. Abbreviations: AKI, acute kidney injury; TyG index, triglyceride-glucose index.
